# The effect of non-neovascular age-related macular degeneration on face recognition performance

**DOI:** 10.1007/s00417-017-3879-3

**Published:** 2018-02-27

**Authors:** Deanna J. Taylor, Nicholas D. Smith, Alison M. Binns, David P. Crabb

**Affiliations:** Division of Optometry and Visual Science, School of Health Sciences, City, University of London, Northampton Square, London, EC1V 0HB UK

**Keywords:** Low vision, Face recognition, Age-related macular degeneration, Geographic atrophy, Visual function, Activities of daily living

## Abstract

**Purpose:**

There is a well-established research base surrounding face recognition in patients with age-related macular degeneration (AMD). However, much of this existing research does not differentiate between results obtained for ‘wet’ AMD and ‘dry’ AMD. Here, we test the hypothesis that face recognition performance is worse in patients with dry AMD compared with visually healthy peers.

**Methods:**

Patients (>60 years of age, logMAR binocular visual acuity 0.7 or better) with dry AMD of varying severity and visually healthy age-related peers (controls) completed a modified version of the Cambridge Face Memory Test (CFMT). Percentage of correctly identified faces was used as an outcome measure for performance for each participant. A 90% normative reference limit was generated from the distribution of CFMT scores recorded in the visually healthy controls. Scores for AMD participants were then specifically compared to this limit, and comparisons between average scores in the AMD severity groups were investigated.

**Results:**

Thirty patients (median [interquartile range] age of 76 [70, 79] years) and 34 controls (median age of 70 [64, 75] years) were examined. Four, seventeen and nine patients were classified as having early, intermediate and late AMD (geographic atrophy) respectively. Five (17%) patients recorded a face recognition performance worse than the 90% limit (Fisher’s exact test, *p* = 0.46) set by controls; four of these had geographic atrophy. Patients with geographic atrophy identified fewer faces on average (±SD) (61% ± 22%) than those with early and intermediate AMD (75 ± 11%) and controls (74% ± 11%).

**Conclusions:**

People with dry AMD may not suffer from problems with face recognition until the disease is in its later stages; those with late AMD (geographic atrophy) are likely to have difficulty recognising faces. The results from this study should influence the management and expectations of patients with dry AMD in both community practice and hospital clinics.

## Introduction

Face recognition is an important daily activity. We are believed to spend more time looking at faces than any other complex visual stimuli, and this is central to social interactions [1]. Difficulties with face recognition can lead to embarrassment and anxiety in social situations, which in turn can lead to social isolation [2]. People with age-related macular degeneration (AMD) have difficulty with different aspects of face recognition. For example, in a survey of 30 people with bilateral AMD, all but one reported difficulty recognising familiar faces on the street; a third of these felt embarrassment as a result [3]. In the same study, over half of respondents reported missing things in conversation because of an inability to make out facial expressions. These patient-reported data are corroborated by performance-based research studies. For example, viewing distances required for recognising faces were found to be shorter on average for people with AMD than those without [4]; moreover, the ability to determine whether or not a face is expressive has been reported to be closely related to near reading acuity [3]. In another study [5], only 26% of 100 people with AMD were able to correctly identify the facial expression in four photographs of people depicting feelings such as happiness, sadness and tiredness; performance in this task was related to visual acuity (VA) in the participants, with those with poorest VA performing particularly badly.

Age-related macular degeneration may be divided into various stages. Early and intermediate [iAMD] AMD are characterised by yellow/white deposits (drusen) beneath the retinal pigment epithelium and/or areas of focal hyperpigmentation or hypopigmentation. Later stages may take one of two forms: neovascular AMD (nAMD), characterised by growth of new blood vessels beneath the retina with a tendency to leak, causing sudden vision loss; or geographic atrophy (GA), characterised by sharply demarcated areas of hypopigmentation caused by atrophy, causing more insidious vision loss [6, 7]. Neovascular AMD is often referred to as ‘wet’, whilst non-neovascular AMD (i.e. early and intermediate AMD and GA) may also be known as ‘dry’ AMD, and constitutes about 90% of diagnosed cases of AMD [8].

Dry and wet AMD have been reported to differ in both their functional [9] and psychological effects [10, 11]. There is a growing interest in characterising the clinical features of different stages of dry AMD, particularly with respect to determining meaningful end points for clinical trials [12]. This interest is timely, as there are several potential therapies for dry AMD currently reaching the stage of phase III randomised clinical trials (RCTs) [12]. Understanding the functional ability associated with each stage of dry AMD is a key element of this characterisation of dry AMD progression. Previous research on face recognition in AMD, however, has largely focused on people with wet AMD or has not differentiated between patients with wet and dry AMD [3, 5, 13]. The aim of this study, therefore, was to investigate face recognition performance in patients with dry AMD of varying severity compared with visually healthy peers.

## Methods

People with dry AMD were recruited from Moorfields Eye Hospital Trust, London, optometrists local to the university and the membership of the Macular Society (https://www.macularsociety.org/). Patients were required to be ≥60 years of age, have sufficiently clear ocular media (grade < 3 on the Lens Opacities Classification System III grading scale [14]), have adequate pupillary dilation and fixation to allow quality fundus imaging, and to have dry AMD (early/intermediate/late) in their better-seeing eye (assessed by visual acuity [VA]). Fellow eyes of patients were permitted to be of any AMD status. Binocular VA of logMAR 0.7 or better (Snellen equivalent of 6/30) was required. Patients were excluded if they had nAMD in their better-seeing eye, had any ocular or systemic disease that could affect visual function or history of medication known to affect visual function (e.g. tamoxifen or chloroquine), high risk of angle closure during pupillary dilation (Van Herick < grade 2, history of angle closure or experience of prodromal symptoms of angle closure). In addition, patients were required to pass an abridged version of the Mini-Mental State Examination [15] and to have sufficient knowledge of the English language to carry out history and symptoms questioning and to understand test instructions.

Age-related controls with healthy vision were recruited from the City Sight Optometry Clinic at City, University of London. People attending this clinic for eye examinations are given the option to agree to be contacted if they wish to be recruited for research studies. Eligibility criteria for controls were the same as for AMD patients, except participants were required to have no AMD (or any other eye disease) in either eye, and monocular VA of logMAR 0.3 (6/12) or better.

The study was approved by a National Health Service Research Ethics Committee and was conducted according to the tenets of the Declaration of Helsinki. Written informed consent was obtained from each participant before examination. Participant information was anonymised before being entered into a secure computer database.

### Clinical tests

Structured history and symptoms were taken. Best-corrected VA was tested using the Early Treatment Diabetic Retinopathy Study (ETDRS) chart (this was scored per letter, and a logMAR score was assigned), and contrast sensitivity (CS) with the Pelli-Robson chart (this was also scored per letter, and a logCS score was assigned). The Van Herick technique was used to assess the anterior chamber angle. Dilated fundus examination was conducted. Lens clarity was graded using a slit lamp biomicroscope, according to the Lens Opacities Classification System III grading scale [14]. Digital colour fundus photographs were obtained, and these were used to classify and grade AMD status by the better-seeing eye (determined by VA) as early, intermediate, or late according to the Beckman classification scale. [6] Spectral-domain optical coherence tomography (OCT) and fundus autofluorescence images were also taken; these, along with slit lamp indirect ophthalmoscopy, were used to support results obtained using colour fundus photographs—for example, OCT to confirm the presence of nAMD, or fundus autofluorescence to confirm the presence of GA.

### Testing procedure

Face recognition was measured binocularly using a modified version of the Cambridge Face Memory Test (CFMT) [16] incorporating eye tracking used in our previous research studies [17, 18]. This was displayed on a 22-inch monitor (Iiyama Vision Master PRO 514; Iiyama Corp., Tokyo, Japan; 1600 × 1200 pixels at 100 Hz). The monitor was placed 60 cm from participants (viewing position was fixed using a head and chin rest), subtending a visual angle of 36.9° horizontally and 28.1° vertically. Images were displayed at an average luminance of 4.29 cd/m^2^ (SD, 1.16). On average, the faces subtended 7.4° horizontally and 11.1° vertically. The average half-angle of faces was 3.7° (equivalent of 6.5 cm width half-face). This is comparable to the size of a face viewed in the real world at approximately 1 m. Optimal refractive correction for the viewing distance was determined by the operator (an optometrist; DJT) prior to testing, and participants all wore this correction mounted in a trial frame. This ensured that any effects caused by frame edges and lens size would be equivalent for each participant.

**Fig. 1:**
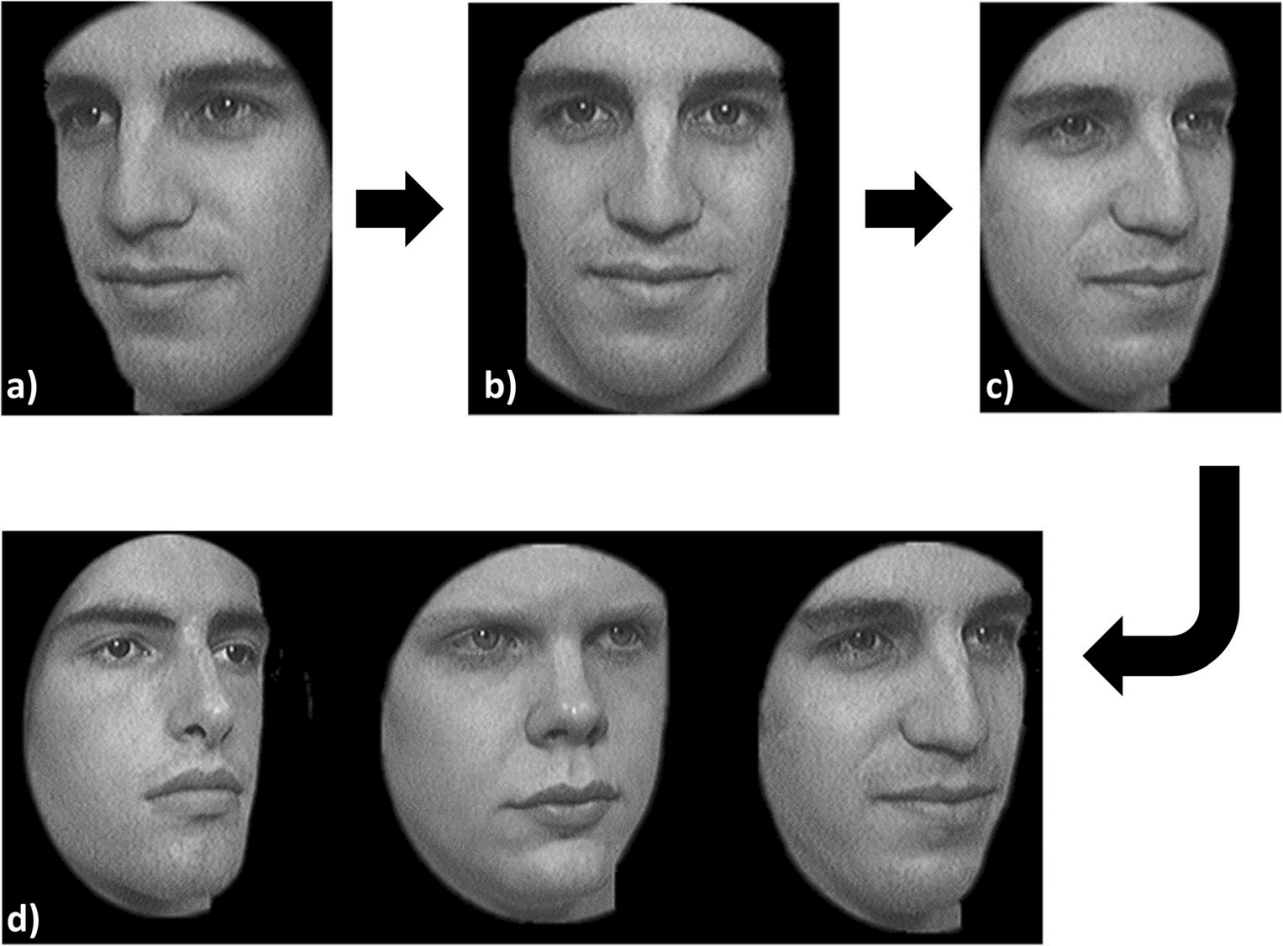
An example task from the selection phase of the Cambridge Face Memory Test (CFMT). Participants were asked to familiarise themselves with a face, shown from three different viewpoints (a, b and c). Participants were then asked to tell the operator which face matched the one they had just viewed (d). Image adapted from Duchaine et al. (2006) [16] with permission from Elsevier

Instructions for the test were both written in large print on the computer screen and given verbally. Trials involved a viewing phase during which participants were shown a series of faces (front and right and left side views) for 3 s per view, and a selection phase during which participants were given a forced-choice task of selecting which face from a set of three matched the one they had just viewed. Responses were keyed in by the operator (DJT). Participants were allowed unlimited time during the ‘selection phase’. Participants completed six trials in this manner (see Fig. 1).

Next, a montage of the six faces learnt during the preceding trials was shown (the ‘review phase’), and participants were asked to study them for 20 s. Recognition of these six faces was then tested by showing participant sets of three faces and requiring them to select the one they had seen before (forced choice again). Overall, participants completed 51 trials.

### Analysis

The percentage of correctly identified faces across the 51 trials was used as the performance outcome measure (FR score) for each participant. A 90% normative reference limit was generated from the distribution of ranked scores recorded in the visually healthy controls. This limit was estimated by a direct percentile method [19]. Scores for AMD participants were then specifically compared to this limit and comparisons made between groups of patients based on severity of AMD in the better-seeing eye. Scores for AMD participants were then specifically compared to this limit and comparisons made between groups of patients based on severity of AMD in the better-seeing eye. This was explored graphically, and Fisher’s exact test was used to test whether the proportion of AMD patients falling outside this limit differed from the proportion of controls falling outside the limit (10%). Amongst people with GA, the relationship between lesion area (as measured using Spectralis RegionFinder software (Heidelberg Engineering, Heidelberg, Germany) and presence/absence of foveal sparing and FR score were explored, again comparing scores to the normative limit set by controls. Mean scores were also calculated for each AMD severity group, and comparisons were made between groups and to mean scores in the visually healthy controls using one-way analysis of variance (ANOVA) and one-way analysis of covariance (ANCOVA) where applicable. Univariate associations between FR score and self-reported disease duration, VA, CS and age were explored. All statistical analysis was conducted in SPSS version 22 software (IBM Corp., Armonk, NY).

## Results

Thirty participants with AMD (87% female) with a median (interquartile range [IQR]) age of 76 (70–79) years, and 34 visually healthy controls (53% female) with a median age of 70 (64–75) years, took part in our study. The median (IQR) duration of AMD was 4 (2–6) years. Participants were of reasonably good general health (ascertained by structured history and symptoms). The median (IQR) ETDRS corrected binocular logMAR VA was 0.22 (0.18–0.38) and −0.06 (−0.12–0) in patients and controls, respectively. The median (IQR) Pelli-Robson logCS values were 1.65 (1.35–1.95) and 1.95 (1.95–1.95) in patients and controls, respectively.

When stratified according to the Beckman classification for the better-seeing eye (no participants had equal VA between eyes), four, seventeen and nine patients were classified as having early, intermediate and late AMD (GA), respectively [6]. Median (IQR) ETDRS corrected binocular logMAR VA according to AMD stage was 0.20 (0.19–0.26), 0.20 (0.13–0.28), and 0.36 (0.24–0.57) for people with early, intermediate, and late AMD respectively. Table 1 shows AMD classification for fellow eyes.

**Table 1:** AMD severity in fellow eyes according to better-eye status

Classification of better-seeing eye	Fellow eye classification			
	Early AMD, no.	Intermediate AMD, no.	Late AMD (GA), no.	Late AMD (nAMD), no.
Early AMD	4			
Intermediate AMD		11	3	3
Late AMD (GA)			8	1

**Fig. 2:**
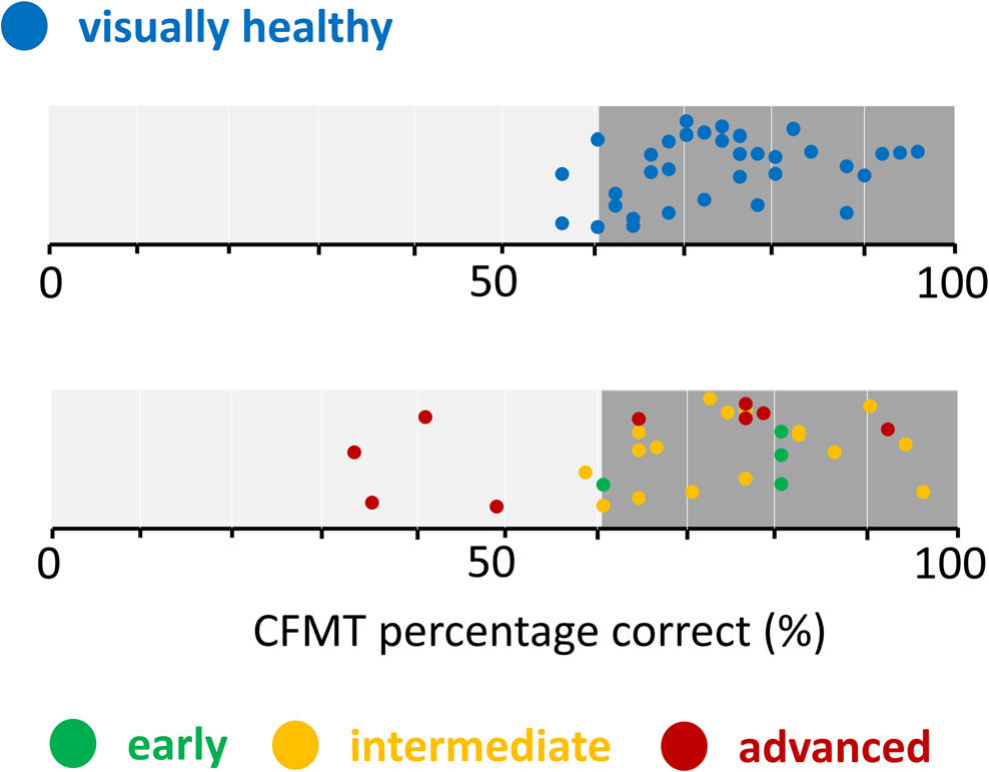
Percentage Cambridge Face Memory Test (CFMT) score for each participant stratified by AMD group. The 90% normative limits set by controls are illustrated by the darker shaded area on the right of both graphs. (Some vertical jitter is added to the plotted points.)

Five (17%) patients recorded a face recognition performance worse than the 90% limit set by controls (Fig. 2). This proportion of AMD patients, as a whole sample, did not differ significantly from the proportion of controls falling outside the limit (Fisher’s exact test, *p* = 0.46). However, four of the five patients falling outside the 90% limit set by the controls had GA.

**Fig. 3:**
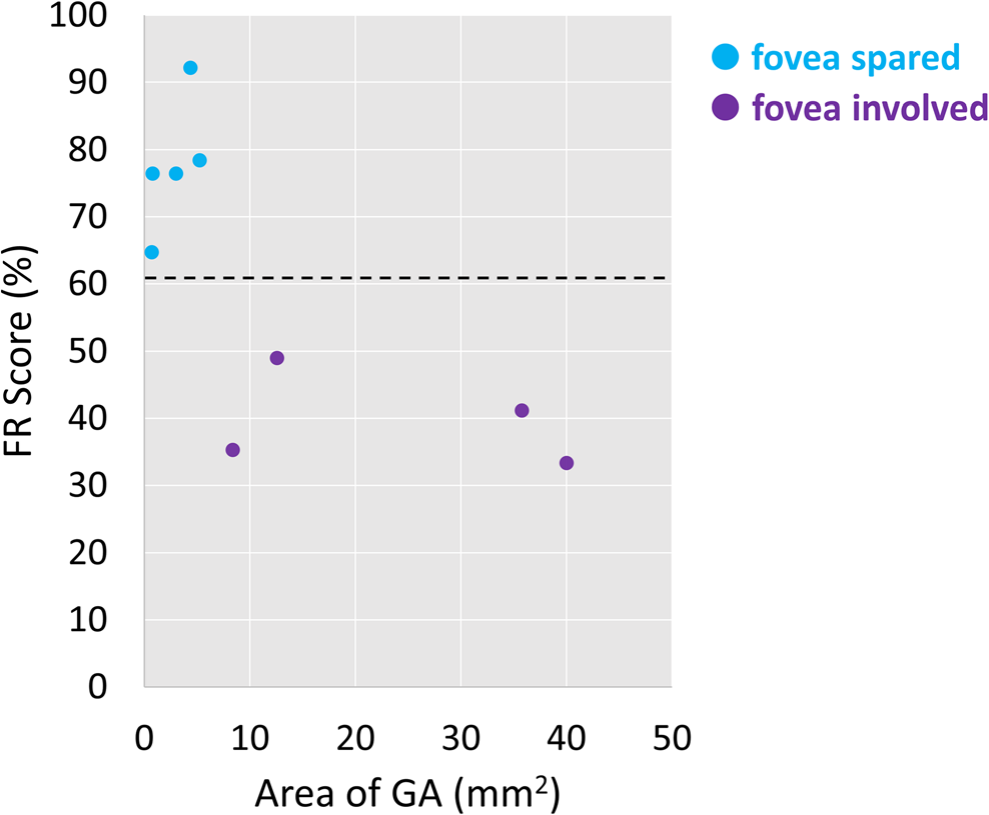
Scatter plot showing the relationship between FR score and GA lesion size amongst participants with dry AMD, colour-coded according to whether foveal sparing was present or not. The dotted black line represents the 90% normative limit derived from the visually healthy controls

Amongst people with GA, those with larger GA lesion area and foveal involvement scored worse on the CFMT than those with smaller lesion area and foveal sparing (Fig. 3). Of the four participants with GA who fell outside the normative limit for FR score set by controls, all had foveal-involving GA and larger lesions than those who fell within the normative limit.

When considering mean effects across the groups, patients with GA identified fewer faces on average (± standard deviation [SD]) (61 ± 22%) than those with early and intermediate (75 ± 11%) AMD and controls (74 ± 11%); this difference was statistically significant (one-way ANOVA, *p* = 0.04). There was a correlation between age and FR score amongst controls (*r* = −0.43, *p* = 0.01), so we corrected our analysis to accommodate for age as a covariate, but the statistical significance of the effect of patients with GA performing worse on CFMT than other groups clearly remained (ANCOVA, *p* = 0.007). No statistically significant differences between groups were observed when participants were grouped according to fellow-eye AMD classification (one-way ANOVA, *p* = 0.39).

**Fig. 4:**
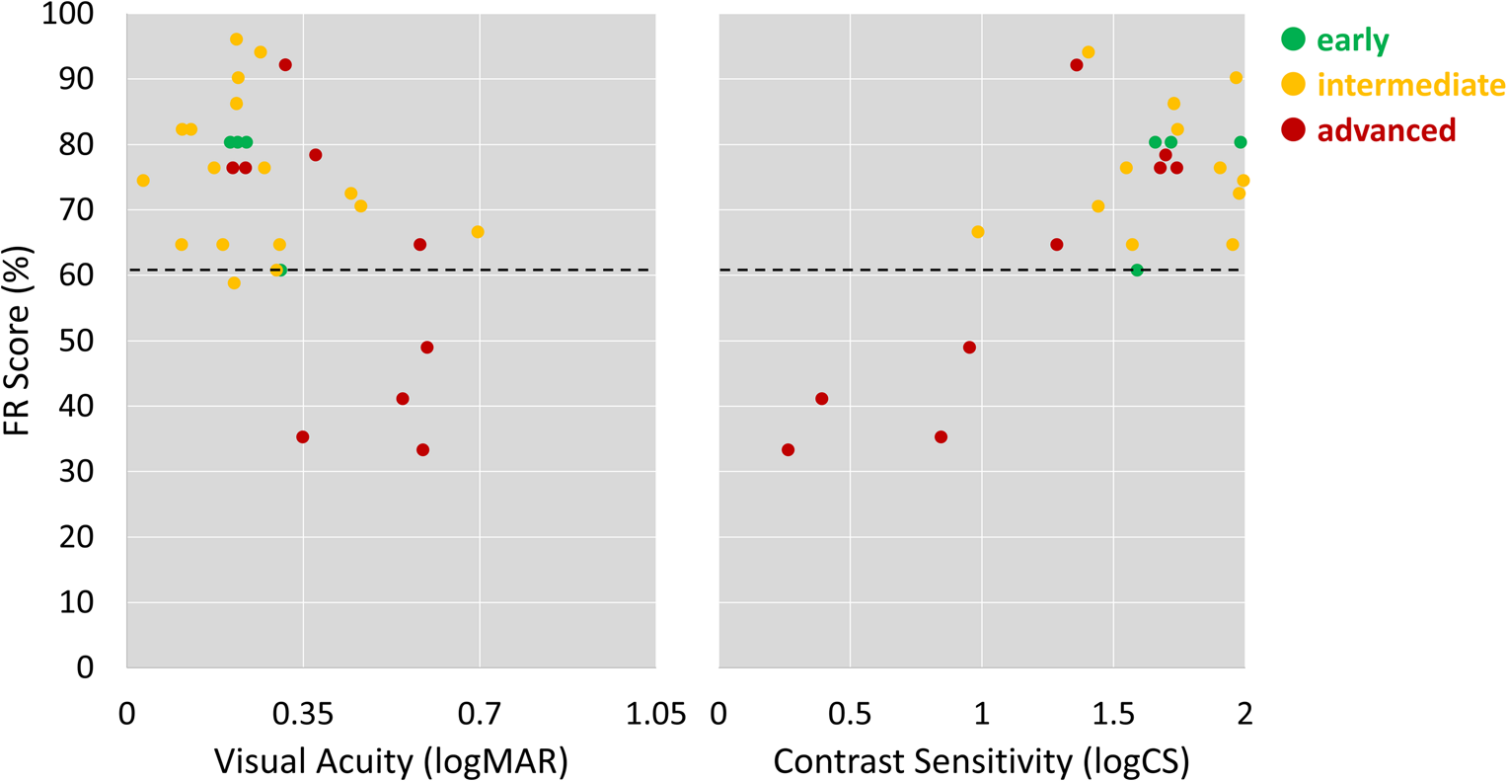
Scatter plots showing the relationship between FR score and contrast sensitivity and visual acuity amongst participants with dry AMD, colour-coded according to AMD classification. The dotted black line represents the 90% normative limit derived from the visually healthy controls. (Some horizontal jitter is added to the plotted points to allow visualisation of overlapping data points.)

Amongst patients, there was a strong statistically significant correlation between FR score and CS (*r* = 0.63, *p* < 0.001), and a weaker but statistically significant correlation between FR score and VA (*r* = −0.54, *p* = 0.002; Fig. 4). There was no significant correlation between AMD duration and CFMT score (*r* = −0.23, *p* = 0.25).

## Discussion

We studied computer-based face recognition performance in patients with a range of severities of dry AMD compared with visually healthy peers. It is well documented that people with AMD have difficulty with face recognition. Our study adds to this knowledge, because it is the first study to document face recognition performance specifically in people with dry AMD. Moreover, we compare FR performance, using a validated test [16], in people with different stages of AMD, as classified by a widely used grading scale [6]. Our results indicate that people with early or intermediate dry AMD perform as well as visually healthy peers on a controlled face recognition task. Therefore, patients with dry AMD may not typically suffer from problems with recognising faces until the disease is in its later stages; those with late AMD (GA) in their least affected eye are likely to have difficulty recognising faces.

Our results are most comparable with a previous study we conducted in people with glaucoma. In our previous work in glaucoma using the CFMT, patients with mild and moderate glaucomatous visual field loss [20] were found to have similar face recognition performance to those without, whilst those with advanced glaucomatous visual field loss were found to have worse face recognition ability. Similarly, in our current study, we report that patients with advanced AMD (GA) have poorer face recognition ability, on average, than those with early and intermediate AMD and those without AMD. However, participants with GA in this study scored worse on average (mean ± SD [61% ± 22%]) than those with advanced glaucomatous visual field loss in our previous study (66% ± 15%), indicating that patients with advanced dry AMD may have greater difficulty with face recognition than those with advanced glaucoma.

The wide variation in FR scores across our results agrees with previous work on face recognition in AMD. Barnes et al. (2011) [13] tested a group of people with AMD with visual characteristics similar to those of our participants (median logMAR VA of 0.20 and CS of 1.55). A wide variability in FR scores was noted, although people with AMD had poorer face recognition on average than did visually healthy controls. Classification and stage of AMD was not reported. A large-scale US-based survey of people with AMD [21] reported a fairly even spread across response options for two of the three items on the Daily Living Tasks Dependent on Vision (DLTV) Questionnaire relating to face recognition. These items described the difficulty experienced in distinguishing a person’s features across a room and across the street. The third item on this questionnaire relating to face recognition asked respondents about difficulty in distinguishing a person’s features at arm’s length. A much higher proportion of respondents (61%) reported ‘no difficulty at all’ for this item compared to the other items; this is relevant to our findings because this item likely reflects difficulty under a task condition most similar to that presented by the CFMT (comparable to viewing a face at 1 m). However, further comparison with our study is limited by the fact that the survey study did not report AMD stage or classification.

We showed a strong association between worsening FR performance and worsening of measured CS in patients with dry AMD. Current evidence surrounding the relationship between FR performance and CS in AMD is conflicting. Some studies [3, 4] report a weak relationship, whilst others report a strong relationship [13, 22]. Other papers have suggested that CS is a more useful indicator of performance of everyday activities [5], mobility [23, 24] and quality of life [25] in people with AMD than other traditional measures of visual function, certainly more than visual acuity alone. However, in a study investigating clinical tests perceived as most and least important by ophthalmologists (albeit almost 20 years ago), CS was consistently rated as one of the least important clinical tests of vision [26]. Our study not only adds to the body of evidence supporting the relationship between FR performance and CS in AMD, but also supports the suggestion that CS may be a more valuable predictor of real-world visual performance than high-contrast VA alone. Median logMAR VA values for participants with early and intermediate AMD (each 0.20) were found to be worse than those of controls (−0.06) in this study. This aligns with VA findings from previous research involving individuals at a similar disease stage [27, 28]. We have also shown that foveal involvement of GA and GA lesion area may be useful predictors of face recognition performance. This implies that the ability to accurately recognise faces is highly dependent on the fovea remaining intact, and provides further evidence to support the development of treatments which may halt or slow the progression of GA before the fovea is affected, such as those currently undergoing clinical trials [12].

Our study has limitations. Although all participants were screened for cognitive defects, it is possible that subtle differences in cognitive ability between participants may have affected the results. Another limitation of this work—and indeed of most face recognition testing—is the questionability of its real-world applicability. The CFMT was chosen for this work because of its strengths compared with other face recognition tests available at the time of testing, its wide use and acceptability (over 300 citations in peer-reviewed literature), its reliability (previous research has consistently confirmed the reliability of the CFMT [Cronbach’s α > 0.8] [29–31]), and specifically its previous use in testing face recognition in eye disease [17, 18]. However, previous research (using different testing methodology) has found a poor relationship between perceived face recognition ability (self-reported difficulties in face recognition reported by almost all participants in the particular study) and performance on a face recognition task [3]. A number of theories attempt to explain these discrepancies. They may occur as a result of differing conditions in the real world, for example, differences in luminance at different times of day and indoors versus outdoors, and differences in viewing distance [32, 33]. Current face recognition testing modalities may not be sensitive enough to detect subtle differences in face recognition ability across people with eye disease of varying severity. A newer test [34] claims to be potentially more sensitive to subtle differences in face discrimination ability than other tests, including the CFMT. Future work might test this further.

There are other potential limitations to our findings. The patients with AMD in our study were very slightly older on average than their visually healthy peers (the 95% confidence interval for mean difference in age was 2–8 years.) However, we corrected our results for this, and it made no difference in our findings. Finally, the fact that 87% of our participants with AMD were female, whilst the control group was only 53% female, might be seen as a limitation. However, the CFMT was designed specifically with male faces, as opposed to female faces or a mixture, because men and women have been shown to exhibit equal FR performance for men’s faces. [16] Therefore, we do not believe that this has influenced the results of our study.

An easily administered and shortened version of the CFMT face recognition task based on our work might have a role as an outcome measure for clinical trials. Our results suggest that the test would not be sensitive to changes in the early and intermediate stages of AMD, but might spotlight a meaningful functional end point when patients develop GA. Results from this type of test are likely to be more meaningful to patients than traditional outcome measures such as letter charts, where changes are often imperceptible to the patient. Development of such a test is the subject of our future work.

To conclude, people with dry AMD may not suffer from problems with face recognition until the disease is in its later stages; those with late AMD (GA), particularly those with larger areas of atrophy involving the fovea and those with significantly reduced CS and (to a lesser extent) visual acuity, are likely to have difficulty recognising faces. This could have important implications for patients, especially when coupled with other problems associated with age-related macular degeneration, for example, difficulties and fears surrounding mobility [35]. The results from this study should influence both management and expectations of patients with dry AMD.
